# Ultraviolet light and polyethylene glycol as environmental cleaning agents to reduce contamination of *Pseudogymnoascus destructans* in bat hibernacula

**DOI:** 10.1371/journal.pone.0341213

**Published:** 2026-01-27

**Authors:** Alyssa J. Stulberg, Tina L. Cheng, Katy L. Parise, Kaleigh J.O. Norquay, Quinn E. Fletcher, Rebecca L. Mau, Daniel L. Lindner, Jeffrey T. Foster, Barrie E. Overton, Winifred F. Frick, Craig K.R. Willis

**Affiliations:** 1 Department of Biology, University of Winnipeg, Winnipeg, Manitoba, Canada; 2 Bat Conservation International, Austin, Texas, United States of America; 3 Pathogen and Microbiome Institute, Northern Arizona University, Flagstaff, Arizona, United States of America; 4 Center for Forest Mycology Research, United States of America Forest Service, Madison, Wisconsin, United States of America; 5 Department of Biological and Health Sciences, Commonwealth University of Pennsylvania, Lock Haven, Pennsylvania, United States of America; 6 Department of Ecology and Evolutionary Biology, University of California, Santa Cruz, California, United States of America; University of Jeddah, SAUDI ARABIA

## Abstract

Pathogens that persist in an environmental reservoir can drive host populations to extinction because host abundance does not limit pathogen survival or reproduction. Fungal pathogens are of particular conservation concern because many fungi are generalists that persist in the environment. One example is *Pseudogymnoascus destructans*, the causative agent of white-nose syndrome (WNS), which has caused severe declines in hibernating bat populations across North America. Treatment of environmental reservoirs could help reduce transmission of *P. destructans*, and thus, reduce bat population declines from WNS. We tested the efficacy of two environmental cleaning agents, ultraviolet-C radiation and polyethylene glycol, in three mines where *P. destructans* established an environmental reservoir and caused declines in winter colony size of hibernating bats in Ontario, Alabama, and Arkansas. We observed considerable variation between sites but, based on our experimental design, treatments did not reduce environmental *P. destructans* prevalence or load and there was no consistent pattern in response to the treatments across mines. More encouragingly, treatments did not impact non-target fungi or bacteria. Our results could reflect aspects of our experimental design, including relatively small treatment cells and the lack of an available assay to assess viability of *P. destructans* from swab samples. Among-site variation we observed, combined with positive results of these treatments in other studies, suggest that site-specific management responses may be important for reducing impacts of white-nose syndrome on bat populations.

## Introduction

The emergence of pathogens that threaten wildlife host populations has generated urgency to develop disease mitigation strategies for management and conservation [1,2]. Disease-induced extinctions are typically rare since many pathogens show density-dependent transmission where pathogen transmission declines with declining host density [3]. However, some pathogens can infect multiple host species or persist in environmental reservoirs, which makes them especially threatening to host populations even potentially driving hosts to extinction [4]. Environmental reservoirs create challenges for disease management because treated or recovered individuals can be re-exposed to the pathogen [5]. For example, experimental treatment of frogs with the fungus *Batrachochytrium dendrobatidis* showed that tadpoles cleared infections when treated with antifungals in the laboratory but were quickly re-infected when returned to *B. dendrobatidis*-positive environments [6]. Thus, in the absence of long-lasting host immunity, targeting environmental reservoirs may be critical for long-term disease management.

White-nose syndrome (WNS), caused by the fungus *Pseudogymnoascus destructans*, has caused site-level declines of greater than 90% in several species of hibernating bats since its introduction to North America [7]. *Pseudogymnoascus destructans* is cold-tolerant and infects the skin of hibernating bats, causing increased energy expenditure during hibernation and premature depletion of winter fat reserves [8–10]. *P. destructans* can persist without hosts in environmental reservoirs within hibernacula [8,11,12] and the environmental reservoir has pronounced implications for short and long-term survival of bats. Individuals might survive WNS during winter, and recover during summer, but become reinfected when returning to *P. destructans*-positive hibernacula in the fall [13–15]. Even following large population declines or extirpation, where bat-to-bat transmission has been reduced or eliminated, the environmental reservoir will continue to expose bats to *P. destructans* and reduce the chance for them to recolonize hibernacula [13–15].

Given the importance of the environmental reservoir to *P. destructans* dynamics [14], strategies that reduce *P. destructans* in the environment could be beneficial for management [16,17]. In particular, if treatments could be applied during summer, when bats are absent from hibernacula, treatment applications would not disturb hibernating bats, which would be preferable for populations that are persisting but still threatened by WNS [16]. Environmental treatments have shown promise for management of other wildlife pathogens with environmental reservoirs, such as alteration of salt concentrations in water bodies to manage chytridiomycosis in Australian wetlands [18] and have recently shown promise for WNS management [16,17,19].

Environmental treatments for WNS have potential, but come with risks, especially if treatment agents indiscriminately target bacteria and other fungi. For example, in the short-term, broad-acting antimicrobials would almost certainly be damaging for microbial communities. Over the longer term, these agents might select for microorganisms/strains with antimicrobial resistance, as observed after antifungal treatments designed to protect Palaeolithic cave paintings [20]. Environmental treatments could also influence microbial community dynamics in unexpected ways. A treatment that inhibits but does not eradicate *P. destructans*, while also impacting *P. destructans*’ competitors, could ultimately cause an increase in *P. destructans* prevalence because of competitive release, as found for pathogenic bacteria following antibiotic treatment in brook trout (*Salvelinus fontinalis*) [21]. Effects of hibernaculum-wide treatments could also depend on initial microbial community composition or other site-specific factors, meaning that effectiveness and non-target impacts of treatments could vary between hibernacula. Therefore, an ideal treatment would: (1) be consistently effective at reducing *P. destructans* abundance across sites; (2) be persistently effective over time to reduce the need for multiple applications; and (3) have minimal impacts on non-target biota.

Numerous biological and chemical agents are at different stages of testing and development for application as anti-*P. destructans* treatment agents on bats and/or substrates [reviewed by 15]. We selected two agents, polyethylene glycol (PEG) and ultraviolet-C (UV-C) radiation, that we expected to be safe for bats and effective at reducing *P. destructans* growth with minimal non-target effects. PEG is an antifungal agent often used to manage fungal infections in agriculture [22] and medicine [23]. Most recently, in experiments with captive bats, PEG was associated with reduced transmission of *P. destructans* from hibernacula to bats, and was associated with reduced *P. destructans* load and infection severity in free-ranging bats [17]. PEG does not kill fungi but instead impedes growth of some fungal species via its effects on matric potential (i.e., surface tension of water) in the fungal growth substrate. More negative matric potentials correspond with increased surface tension which reduces water available for fungal growth [24]. Furthermore, environmental PEG application would minimally impact non-target biota because PEG has low toxicity for animals [25] and, while many soil fungi are susceptible to PEG-induced matric stress [24], the matric stress required for inhibition varies by species and PEG does not affect non-fungal microbes. Matric potentials of −5 MPa inhibit mycelium growth and spore germination of *P. destructans* [26] and this effect could be specific to *P. destructans* among cave-inhabiting fungi.

Another option for environmental treatment against *P. destructans* is UV radiation. UV light is widely used to disinfect food, water, and air, but efficacy depends on both wavelength and dose [27]. *P. destructans* is not harmed by UV-A light but is sensitive to higher energy UV-B (312 nm) and UV-C light (254 nm) [28]. A 10 mJ/cm^2^ dose of UV-C light resulted in < 1% survival of *P. destructans*, with minimal effects on non-target biota [19,28]. *P. destructans* lacks UVE1, an enzyme vital in repairing UV-induced DNA damage [28]. UVE1 is present in most other fungi, including non-pathogenic *Pseudogymnoascus* species [28]. *P. destructans* does possess DNA photolyases, another class of DNA repair enzymes, but their activation requires light so they are unlikely to be effective in underground environments. Kwait et al. [19] demonstrated the efficacy of UV-C as a cleaning agent in the laboratory but efficacy to treat the environmental *P. destructans* reservoir in hibernacula has not yet been published.

We conducted a side-by-side comparison of PEG and UV-C treatment of the *P. destructans* reservoir in hibernacula. Specifically, we tested the hypothesis that UV-C light and PEG are effective, long-lasting, and safe for reducing *P. destructans* on bat hibernaculum substrates. To control for potential effects of environmental conditions on *P. destructans* growth and efficacy of treatments, we designed the study with *in situ* replication by creating 20 cm diameter circular areas (hereafter ‘cells’) replicated 120 times for each treatment, within three WNS-positive bat hibernacula across a large portion of the range of WNS (Alabama, Arkansas and Ontario). Given the positive results from previous studies [17,19], we predicted that: 1) Cells treated with PEG and UV-C would have reduced prevalence and loads of *P. destructans* relative to control cells; 2) A one-time treatment in early fall, before hibernation onset, would have lasting effects throughout winter, avoiding the need for multiple treatments which could disturb hibernating bats; and 3) PEG and UV-C radiation would have minimal impacts on non-target microbiota.

## Materials and methods

All methods were approved by the University of Winnipeg Animal Care Committee, Ontario Ministry of Natural Resources (Wildlife Scientific Collector’s Authorization Permit: 1089801), Alabama Department of Conservation and Natural Resources: Wildlife and Freshwater Fisheries Division, and the Arkansas Game and Fish Commission. The experiments took place in three abandoned mines, which were at the northern- and southernmost frontiers of *P. destructans* spread and WNS establishment at the time of our experiment (i.e., 2018–2019). These sites included: Richard Lake Mine, a former uranium mine near Kenora, Ontario in Treaty 3 territory (49.7706°N, −94.4895°E, hereafter the Ontario site) at which *P. destructans* was first detected in January of 2017, an unused manganese mine in the Ouachita National Forest in Arkansas (34.479194°N, −94.1583°E, the Arkansas site) where WNS was confirmed in 2014, and an unused iron mine in the Ruffner Mountain Nature Preserve in Birmingham, Alabama (33.5599°N, −86.7078°E, the Alabama site) where WNS was confirmed in 2017. We chose mines for the experiment so we could replicate natural conditions for *P. destructans* growth without disrupting poorly understood and potentially sensitive biota that live in natural caves. Sites were chosen based on knowledge of pre-WNS colonies of at least hundreds of *Myotis lucifugus* and/or *Perimyotis subflavus*, two species that have experienced major declines from WNS across their range [7], the presence of *P. destructans* and WNS for at least eight months prior to our experiment [29], and the suitability of these sites to construct barriers to exclude bats from experimental areas. We do not have precise pre- and post-WNS count data for these sites but all three housed some bats (outside of our exclosures) during the experiment despite dramatic declines in numbers of bats between detection of *P. destructans*/WNS and the winter of our experiment. All sites were also similar in terms of humidity/moisture levels (see below) and had qualitatively low levels of organic material. We constructed barriers to exclude bats from the sections of each hibernaculum where we established the replicated treatment cells in September and October 2018 prior to the onset of hibernation to ensure bats did not enter and potentially spread *P. destructans* during the experiment. While excluding bats from part of a hibernaculum is not a real-world scenario replicating how treatments might be applied at scale, in this experiment, we aimed to eliminate potential variation in *P. destructans* caused by bats shedding fungus while testing the efficacy of the environmental treatments. Barriers were made of wood framing, 1.27 cm wire mesh, and insulating spray foam (Great Stuff, Gaps and Cracks Foam Sealant, model #99108824) with doors to allow secure entrance and exit by researchers.

We defined circular 20 cm diameter cells on the walls and ceilings of the mine within the experimental zone at each site. We grouped cells in blocks of four, with one cell in each block corresponding to each treatment: UV-C light, PEG solution, broad-based decontamination control (i.e., 90% isopropyl alcohol), and control (no treatment). We colour-coded each cell with lumber crayon, the colour of which remained visible on the rock surface throughout the study. Each block of four cells was arranged in a horizontal row with 40 cm between cells to minimize runoff and contamination between cells. Blocks were equally divided between the walls and ceiling of each mine, as previous research has shown that *P. destructans* prevalence and loads can differ on ceilings compared to vertical surfaces [30]. Within each block, we randomized the order of our four treatments and each treatment was replicated 30 times for a total of 120 cells per mine. We selected 120 treatment cells to ensure that we would have a large enough sample size of cells with *P. destructans* present at the start of the experiment in sites with low or variable prevalence. At every site, we measured surface temperature within each cell with a digital infrared thermometer (Fluke 62 Max) and categorically assessed moisture level (dry, wet, or dripping) of each cell before treatment and subsequent sampling. Our use of replication inside each hibernaculum allowed us to rigorously control for environmental variation that could, otherwise, have influenced growth of *P. destructans* or other microbes, and the potential efficacy of treatments.

### Sample collection and treatments

Treatments were applied between 25 September and 31 October 2018 beginning with Ontario and ending with Arkansas. Based on long-term acoustic data collected as part of another study (Cheng and Frick, unpublished data), bats hibernate from early October to mid-May in the Ontario site, from late October to early March in the Arkansas site, and from early November to late March in the Alabama site. Thus, treatments were applied within approximately 1–3 weeks of when bats would normally begin hibernation at these sites. Immediately prior to treatment application, we swabbed every cell once (Fisherbrand Polyester-Tipped Applicators) to confirm the presence of *P. destructans*, quantify *P. destructans* load, and determine pre-treatment microbiome community structure. Swabs were dipped in sterile water and then run across the substrate surface in a 20 cm transect that bisected each circular cell in an ‘X’ pattern. We stored swabs in RNAlater to preserve the DNA at room temperature until processing. We applied treatments up to two days after barriers were installed and all cells in the experimental area had been sampled.

For the decontamination control we applied 3.5 mL of 90% isopropyl alcohol to treatment cells using a spray bottle, saturating the substrate but not creating runoff. This volume was determined by counting the number of times isopropyl could be sprayed in a 20 cm diameter circle on a hibernaculum surface before running down the wall. We quantified this volume by directing the same number of sprays into a graduated cylinder. We sprayed the solution ~15 cm from hibernaculum walls and ceiling, with each spray aimed at a different quadrant within the treatment cell to ensure full coverage. This treatment acted as our decontamination control as it is a known disinfecting agent for *P. destructans* [33,34]. We left control cells untreated, which allowed us to measure undisturbed dynamics of *P. destructans* growth and microbiome community structure within each site.

For the UV-C treatment we applied 100 mJ/cm^2^ of UV-C light (254 nm) to each UV-C treatment cell with a Sterilaire Lamp, Model XX-15S, 15W (Analytik Jena, Jena, Germany). This exposure level was four times higher than that required to kill *P. destructans* spores in the laboratory (Palmer et al. 2018). We used this relatively high dose to reduce effects of shadows from the irregular surface of the walls and ceiling in the mine. UV-C exposure lasted ~10 seconds per cell. However, due to the potential for the UV lamp to accumulate dust and grime, and for lamp intensity to decline as batteries ran out, we ensured a consistent dose to each cell by measuring the UV-C intensity of the lamp every five cells using a UVX Digital Radiometer and a UVX-25 shortwave sensor (Analytik Jena, Jena, Germany) and then adjusting treatment duration accordingly. We fit a metal cone (25 cm diameter, 22 cm length) to the UV-C light to ensure the distance between UV-C bulbs and substrate was uniform between cells and to block UV-C exposure to nearby cells. The UV-C light was turned on for 30 minutes prior to treatment to allow the bulbs to warm up and reach steady-state intensity.

For PEG treatment cells we applied a solution of PEG with molecular weight 8000 (PEG 8000 – Fisher BioReagents) in deionized water. Since it is unknown how environmental variables might influence the effectiveness of PEG, we used double the effective concentration required to inhibit spore germination and mycelium growth of *P. destructans* [26] using the formula:


$$𝛹=\text{1}.\text{2}\text{9}[PEG{]}^{\text{2}}T-\text{1}\text{4}\text{0}[PEG{]}^{\text{2}}-\text{4}.\text{0}[PEG]$$

where T = temperature (°C) and [PEG] = grams of PEG per ml of water [35]. Since temperature varied between 5–15°C depending on mine location and time of year, we used [PEG] = 0.28 g/mL, which corresponds to matric potentials between −11.6 and −10.6 MPa respectively, which is similar to those found to be effective in a recent field trial [17]. We sprayed 10.25 g of the solution onto each cell from ~30 cm away using a cordless paint sprayer (Graco Ultra Cordless Airless Paint Sprayer, model #17M363, USA) with the speed setting set to one. To ensure a uniform dose we sprayed each cell for two seconds. This formed a sticky, thin film of PEG solution on the substrate without resulting in runoff that could have washed off spores or mycelium.

In addition to pre-treatment swabbing, cells were swabbed three additional times following treatment applications during early winter (7–8 weeks post treatment), late winter (20–21 weeks post treatment), and summer (33–34 weeks post treatment). Sites were sampled in the same order as they were originally treated (i.e., Ontario first and Arkansas last). Transect angle within each cell was shifted by 22.5° every sampling period so that only the very centre of each cell was re-swabbed during subsequent sessions (S1 Fig). We were interested in the potential of our treatments to reduce the prevalence and load of *P. destructans* so we only collected swabs, throughout the experiment, from control and treatment cells that were initially positive for *P. destructans* prior to treatment thus our actual starting sample size of cells in each site was less than the original 120 (S1 Table). During late winter and early summer, we also collected swabs from 30 randomly chosen cells on walls and ceilings outside each bat exclosure to test whether excluding bats influenced temporal changes in *P. destructans* prevalence and load.

### DNA extraction and sequencing

Swabs were shipped on ice to the Pathogen and Microbiome Institute (PMI) of Northern Arizona University, Flagstaff, AZ. We extracted DNA using a DNeasy Blood and Tissue kit (Qiagen Inc) following the manufacturer’s yeast extraction protocol, modified to use a vacuum apparatus in place of centrifuging. Quantitative polymerase chain reaction (qPCR) assays following Muller et al. [36] were used to determine prevalence and loads of *P. destructans* Primers targeted the intergenic spacer (IGS) region of the multicopy rRNA gene complex (forward primer CNL12: (5’ – CTG AAC GCC TCT AAG TCA G – 3’) [37] and reverse primer CNS1: (5’ – GAG ACA AGC ATA TGA CTA CTG – 3’) [38]. The IGS region allows for accurate identification of *P. destructans* due to its high interspecific variation, even among closely related *Pseudogymnoascus* species. To ensure high sensitivity of samples with low fungal quantity, we ran two plate qPCR assays for every swab. Samples were considered positive for *P. destructans* when at least one qPCR run crossed the cycle threshold within 40 cycles. Each 96-well assay plate included three serially diluted positive controls for *P. destructans* and 16 no-template controls (containing reagents but no sample). We estimated fungal load in nanograms for each qPCR run based on the cycle threshold (C_t_) value, using the formula [39]:


$$\text{load}\;(\text{ng})={\text{10}}^{\left((\text{22}.\text{04942}-\text{Ct}\;\text{value})/\text{3}.\text{34789}\right)}$$

We then averaged the load values of duplicate runs to determine the load for each cell, assuming qPCR runs with Ct > 40 cycles had 0 ng of *P. destructans*. To assess non-target effects, we determined microbiome community composition using extracted DNA from the same swabs used for qPCR. This analysis was only conducted for samples from the Ontario site because *P. destructans* prevalence was very low at the other two mines (see Results). We constructed fungal libraries for sequencing by running a two-step PCR following Alvarado et al. [40]. Primers targeted the internal transcribed spacer 2 (ITS2) region of the fungal rRNA gene complex (5.8S-Fun (5’ – AACTTTYRRCAAYGGATCWCT – 3’) and ITS4-Fun (5’ – AGCCTCCGCTTATTGATATGCTTAART – 3’) [41] with attached universal tails. Although IGS is known to be more accurate in distinguishing between *Pseudogymnoascus* species, ITS is the most widely sequenced region among fungal groups and was therefore used to characterize the fungal microbiome. The initial PCR was performed in duplicate in 15 μL reactions consisting of 1X Phusion Green Hot Start II High-Fidelity PCR Master Mix (Thermo Fisher Scientific), 0.2 μM of each forward and reverse primer, 1.5 mM MgCl_2_, and 5 μL template DNA. Each sample plate also included a no-template control. Samples were amplified with the following reaction conditions: initial denaturation at 95°C for 2 min, 25 cycles of denaturation at 95°C for 30 sec, annealing at 55°C for 30 sec, extension at 72°C for 30 sec, and a final extension step of 72°C for 10 min. Duplicate PCR products were combined, checked on a 1% lithium borate gel (250V, 500mA, 15 min), and diluted tenfold. The second round of PCR added unique indexes to each sample with primers that matched the universal tails. The 15 μL reaction included 1X Phusion Green Hot Start II High-Fidelity PCR Master Mix (Thermo Fisher Scientific), 0.1 μM of each forward and reverse primer, 1.5 mM MgCl_2_, and 2 μL template DNA. Reaction conditions were the same as the first round of PCR but were carried out for 20 cycles as opposed to 25. PCR products were checked again with gel electrophoresis, then purified with AMPure XP beads (Beckman Coulter) and normalized to equimolar concentrations using a SequalPrep Normalization kit (Thermo Fisher Scientific). Resulting samples were pooled together at equal concentrations and concentrated using AMPure XP beads. DNA concentrations were quantified using a Qubit Fluorometer (Thermo Fisher Scientific). Samples were sequenced on an Illumina MiSeq instrument using a 2 x 250 cycle v2 kit (Illumina) at the Translational Genomic Research Institute (TGen) North facility in Flagstaff, Arizona, USA.

Bacterial libraries were constructed following a modified Earth Microbiome Project protocol [42]. The 16S rRNA region was amplified using the 515F forward primer (5’ – GTGYCAGCMGCCGCGGTAA – 3’) [43] and the 806R reverse primer (5’ – GGACTACNVGGGTWTCTAAT – 3’) [44] with attached unique indexes. PCRs were conducted in duplicate in 25 μL reactions containing 0.625 U HotStart Ex Taq (Takara Bio USA), 1X Buffer, 0.3 mM dNTPs, 0.4 μM forward and reverse primers, 0.56 mg/mL bovine serum albumin, and 2 μL template DNA. Each sample plate also included a no-template control. Samples were amplified under the following conditions: initial denaturation at 98°C for 2 min, 30 cycles of denaturation at 98°C for 20 sec, annealing at 50°C for 30 sec, extension at 72°C for 45 sec, and a final extension of 72°C for 10 min. Duplicate PCR products were combined and prepared following the same gel electrophoresis, bead purification, normalization, pooling, concentrating, and quantification protocols as described above for the ITS2 amplicons. Samples were sequenced on an Illumina MiSeq instrument using a 2 x 150 cycle v2 kit (Illumina) at the TGen North facility.

### Analyses

To test for effects of treatment types (i.e., UV-C light, PEG, isopropyl alcohol, or control) on *P. destructans* prevalence (*P. destructans* absent [0] vs. present [1] in the cell)*,* we used a generalized linear mixed-effects model with a binomial distribution using the glmer function from package lme4 (v. 1.1.29). To test for effects of treatment type on *P. destructans* load (log_10_ transformed), we used linear mixed-effects models, assuming a Gaussian distribution, using the lmer function (lme4; v. 1.1.29) [45]. We did not treat or subsequently sample from cells for which there was no *P. destructans* detected during the pre-treatment sampling period (n = 10/120 cells from the Ontario site, 85/120 cells from the Alabama site and 78/120 cells from the Arkansas site), because we were interested in how our treatments might reduce *P. destructans* prevalence and load and it would not be possible to reduce levels of these variables with no fungus present. We included all treated and sampled cells in our analysis of *P. destructans* prevalence but, for the analysis of *P. destructans* load, we excluded cells from sampling periods when *P. destructans* load in those cells fell to zero (S1 Table). In both the *P. destructans* prevalence and load analyses, cell ID was included as a random effect to account for re-swabbing the same cells over time. Initial analysis included study site as a random effect to account for non-independence among grouped samples and unmeasured variation among sites, but visual inspection of the data showed a clear influence of site on *P. destructans* growth and treatment results, likely due to variation in site characteristics that we could not identify. Therefore, we analyzed data for each site independently. We fit a global model, which included treatment as a categorical variable (untreated negative control, positive control – isopropyl alcohol, PEG, UV-C), sampling time as a continuous variable (specified as number of days since pre-treatment sampling divided by 10 to facilitate model convergence), and location within the mine (wall vs. ceiling) as fixed effects. We also attempted to include a treatment by sampling time interaction to determine if the influence of treatments varied over time, especially in comparison to the positive (isopropyl alcohol) and negative (untreated) controls. We did not include moisture level in our models because it was confounded with sampling time, as most cells during pre-treatment were “wet” and all cells after this period were “dry”. If the treatment:sampling time interaction was not significant, or the model including this term did not converge, it was removed from the model and a simplified model excluding this term was fit. We tested the significance of all terms in our *P. destructans* prevalence and load analyses using likelihood ratio tests (ANOVA) [46] assuming a χ^2^ distribution and the degrees of freedom equal to the difference in the number of terms in the nested models (nested models fit using maximum likelihood). At some sites, the full *P. destructans* load and prevalence models were singular or would not converge. This could mostly be attributed to low *P. destructans* prevalence (and thus many NA *P. destructans* load values) at some sites during some sampling periods. In these instances, models were reduced and/or the random effect of cell ID was removed.

We analyzed microbial community results using QIIME2 (version 2019.7) [47] an open-source microbiome informatics platform. Primers were trimmed from the paired-end, demultiplexed sequences using cutadapt [48] and reads were quality filtered using DADA2 [49]. ITS2 rRNA sequences were truncated to 230 bp because the length of this gene region can be variable across fungal species. Forward and reverse reads were merged for downstream 16S rRNA analysis, whereas ITS2 rRNA analysis used only forward reads due to high sample loss after merging with low quality reverse reads. Representative sequences were chosen for each operational taxonomic unit (OTU) based on 97% sequence similarity. Taxonomy was assigned using a Naive Bayes classifier trained against the Greengenes 13_8 [50] reference database for bacterial analysis and against the UNITE [51] reference database for fungal analysis. We amplified 13,379,582 bacterial sequences from 377 samples that were then classified into 6,471 OTUs. We also amplified 8,999,806 fungal sequences from 384 samples that were then classified into 6,412 OTUs. Generated OTU and taxonomy tables were exported to R for further analysis. Contaminants were identified using the package decontam (threshold = 0.5) [52] in R, which compares sequence prevalence in negative controls against true samples. There were 14 bacterial taxa identified as contaminants and were subsequently removed from analysis. We also filtered out sequences from the genus *Cellulosimicrobium*, which were by-products of using lyticase for fungal DNA extraction. A total of 2,263,498 bacterial sequences that classified into 6,397 OTUs remained after contaminant removal. For fungal analysis, nine contaminants were identified and removed. We also removed the genus *Pseudogymnoascus* from the data because potential reductions in *P. destructans*, the targeted fungal species, would alter calculations of non-target effects. Since ITS sequencing is not able to reliably distinguish between *Pseudogymnoascus* species, the entire genus was removed. This resulted in 7,891,157 retained sequences that were classified into 6,324 OTUs. Bacterial samples were rarefied to a depth of 1,739 sequences/sample, which retained 21.7% of features in 80.1% of samples. Fungal samples were rarefied to a depth of 9,861 sequences/sample, which retained 37.6% of features in 80.2% of samples.

To determine if treatments influenced microbial diversity in the Ontario site, we used both alpha and beta diversity metrics. We calculated the Shannon Diversity Index for alpha diversity, which includes both species richness (the number of taxa present) and species evenness (relative abundance of taxa). We fit a global linear mixed-effects model to test whether treatments influenced Shannon Diversity Index values for bacteria (log_10_ transformed) and fungi (normally distributed) operational taxonomic units, following the method described above. To assess overall community composition, we calculated unweighted and weighted UniFrac distances in QIIME2, which incorporate phylogenetic relatedness into measurements of dissimilarity. A UniFrac distance of zero implies identical communities, whereas higher UniFrac distances imply less similarity between samples. Principal Coordinate Analysis (PCoA) was used to visualize results. Following visual inspection, we used *qiime longitudinal first-distances* to calculate distance matrices between successive samples from the same cell ID based on unweighted and weighted UniFrac values. We ran linear mixed effect models using outputs from these distance matrices. Treatment and sampling time were included as fixed effects, while random intercepts for cell ID were included by the method.

## Results

### *P. destructans* prevalence and load

**Fig 1:**
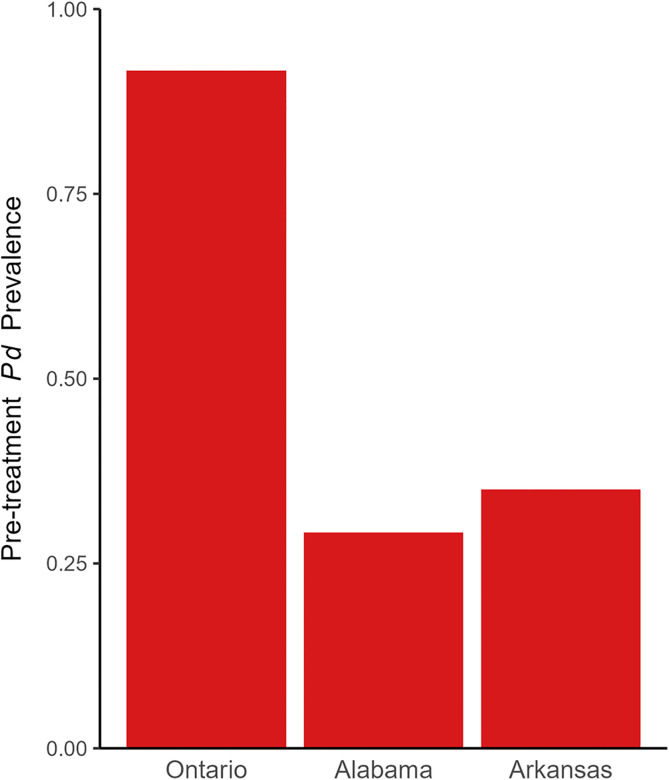
Site-wide *P. destructans* prevalence in Oablentario, Alabama, and Arkansas before treatment application. For each location, n = 120.

**Fig 2:**
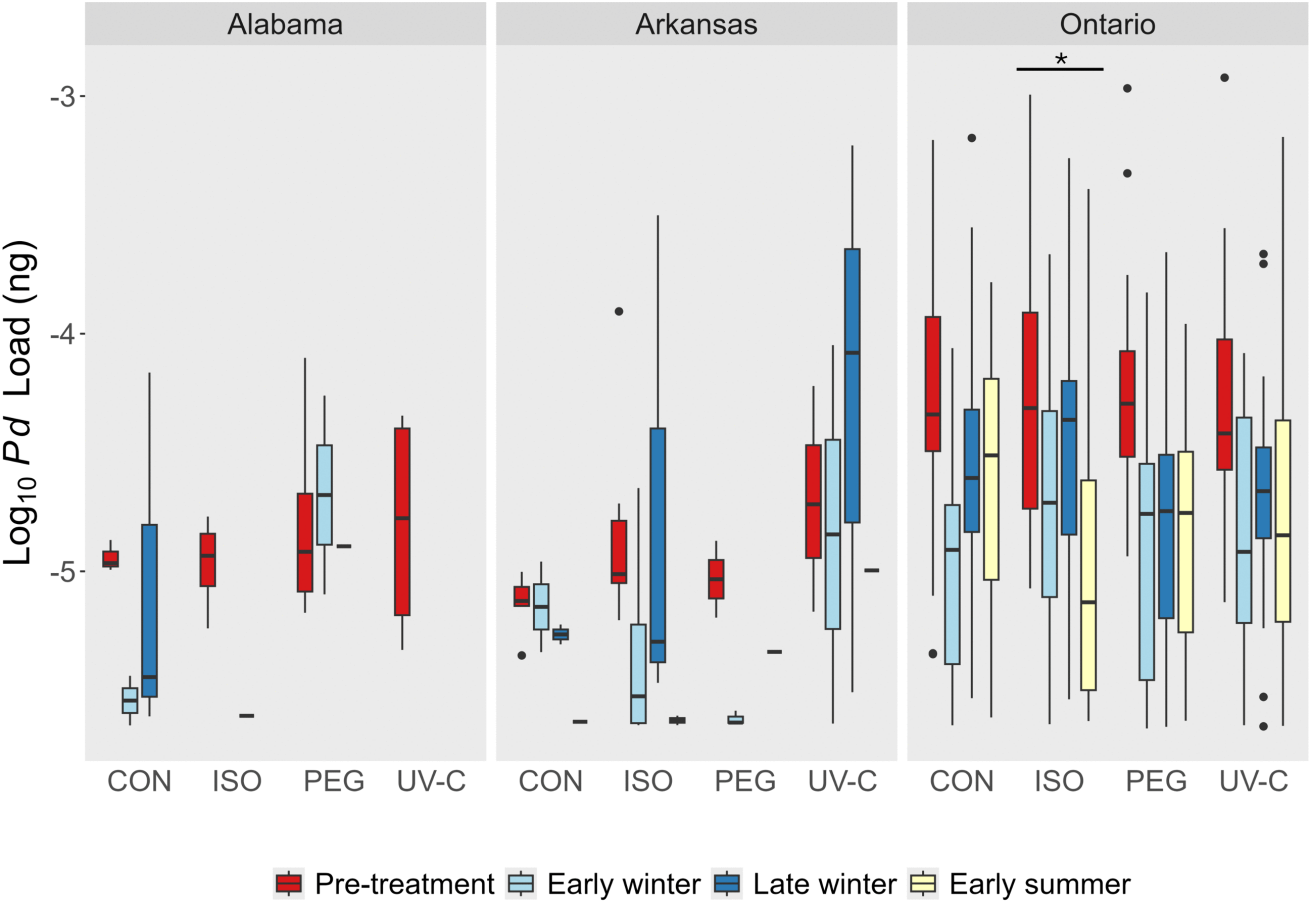
Log_10_ transformed fungal loads of *Pseudogymnoascus destructans* in ng across four treatments during the 2018–2019 hibernation season in the Arkansas, Ontario, and Alabama sites. CON = control, ISO = isopropyl alcohol, PEG = polyethylene glycol, UV-C = ultraviolet-C radiation. Circles represent individual outliers at each treatment-time combination. *P. destructans* was not detected during early summer sampling in the Alabama site. Note that time was treated as a continuous predictor variable in our models (see methods) but was difficult to visualize graphically when combined with treatment so is plotted here as discrete bars for convenience. No treatment effects were significant but the significant interaction between time and treatment (driven by the effect of isopropyl alcohol; see results) is shown by the asterisk. No other treatment or time effects were significant.

**Fig 3:**
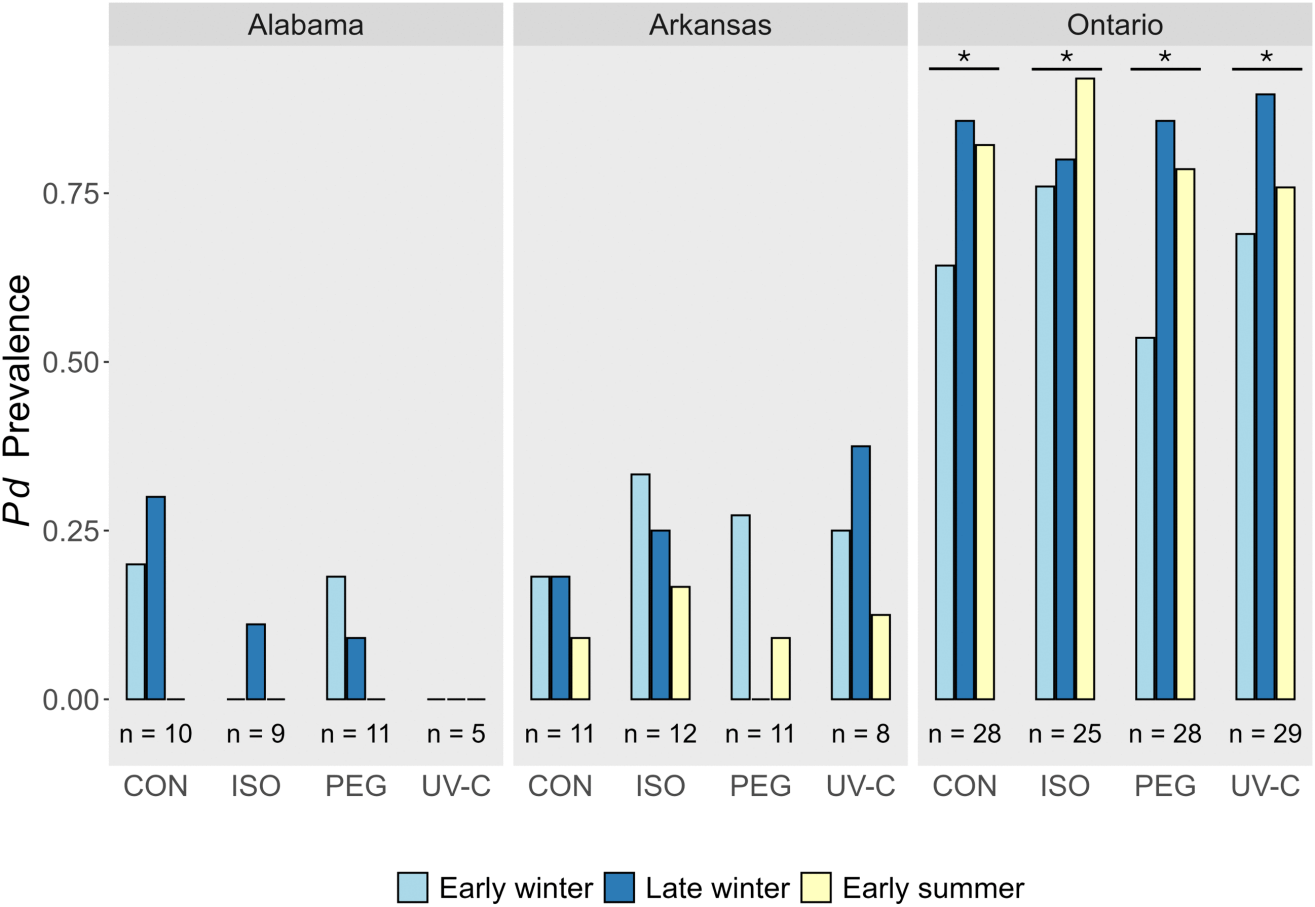
The fraction of sampled 20 cm diameter treatment cells that tested positive for *Pseudogymnoascus destructans.* Each site had 120 cells during the pre-treatment period; however, only cells that were *P. destructans* positive were retained in the experiment, which is reflected in the sample sizes (n=). CON = control, ISO = isopropyl alcohol, PEG = polyethylene glycol, UV-C = ultraviolet-C radiation. Note that time was treated as a continuous predictor variable in our models but was difficult to visualize when combined with treatment so is plotted here as discrete bars for convenience. No treatment effects or interactions were significant but significant effects of sampling time, which only occurred in the Ontario site (see results), are shown by asterisks. The effect of sampling time was not significant in the Alabama or Arkansas sites.

Contrary to our prediction, we found no difference in *P. destructans* prevalence or load between UV-C or PEG-treated cells compared to the negative and positive control cells at any of our sites (Figs 2 and 3). The prevalence of *P. destructans* in cells prior to treatment application in the fall, just before hibernation, varied between sites with 29% in Alabama, 35% in Arkansas, and 92% in Ontario (Fig 1). Excluding cells that were *P. destructans* negative, *P. destructans* load was dramatically higher in the Ontario site in the pre-treatment sampling period (1.04 x 10^−4^ ng, SD = 2.0 x 10^−4^ ng; n = 110) than in the Alabama (1.2 x 10^−5^ ng, SD = 1.5 x 10^−5^ ng; n = 35) or Arkansas (1.0 x 10^−5^ ng; SD = 2.0 x 10^−5^ ng; n = 42) sites (Fig 2). Only cells that were *P. destructans* positive during the pre-treatment period were treated (PEG, UV-C, Isopropyl, Untreated control) and monitored in additional sampling periods. During subsequent sampling periods, there was no difference in *P. destructans* prevalence among any treatment types or controls in the Alabama (χ^2^ = 5.9, df = 3, *P* = 0.12; S2 Table; Fig 3), Arkansas (χ^2^ = 1.7, df = 3, *P* = 0.64; S3 Table; Fig 3) or Ontario sites (χ^2^ = 1.9, df = 3, *P* = 0.59; S4 Table; Fig 3). We found a similar result for *P. destructans* load where *P. destructans* loads in the PEG treated cells did not differ from those in the untreated control cells in Alabama (*F*_1,6_ = 1.5, *P* = 0.26; S5 Table). In Arkansas, we also detected no difference in fungal loads among the treatment types (χ^2^ = 5.2, df = 3, *P* = 0.16; S6 Table). In Ontario, there was a significant treatment by time interaction influencing *P. destructans* loads (χ^2^ = 9.0, df = 3, *P* = 0.03; S7 Table) driven by the stronger decline of *P. destructans* load values in the Isopropyl treatment in the early summer sampling period. *P. destructans* loads were also significantly higher on the ceilings than the walls in the Ontario site (χ^2^ = 11.6, df = 1, *P* = 0.0007; S7 Table).

Restricting our analysis to cells that were positive for *P. destructans* in the pre-treatment period, *P. destructans* prevalence decreased by a greater amount from pre-treatment to early winter in the Alabama and Arkansas sites (pooling all cells: Alabama = 89%; Arkansas = 74%; Figs 1 and 3) as compared to in the Ontario site (pooling all cells: 35%; Figs 1 and 3). Interestingly, there was a strong trend for *P. destructans* prevalence to continue to decrease in the sampling periods following the early winter period in the Alabama (χ^2^ = 3.3, df = 1, *P* = 0.07; S2 Table) and Arkansas sites (χ^2^ = 3.6, df = 1, *P* = 0.06, S3 Table). Conversely, *P. destructans* prevalence in the Ontario site increased over time in the sampling periods after the early winter period (χ^2^ = 9.8, df = 1, *P* = 0.002, S4 Table).

**Fig 4:**
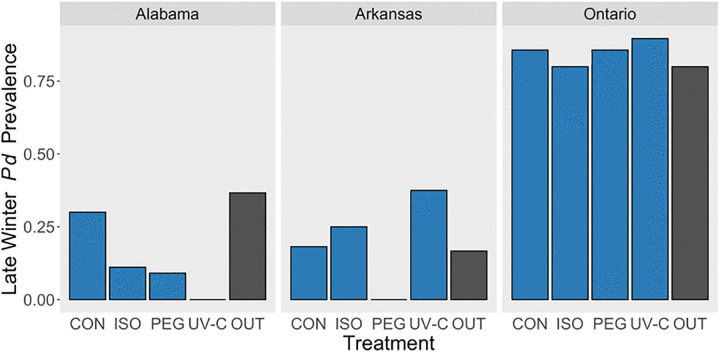
Fraction of sampled cells with *Pseudogymnoascus destructans* present in bat hibernacula sites in Alabama, Arkansas, and Ontario during late winter (February – March 2019). CON = control, ISO = isopropyl alcohol, PEG = polyethylene glycol, UV-C = ultraviolet-C radiation, OUT = randomly selected areas outside the treatment zone where bats were excluded. Areas sampled where bats were present were not significantly different from untreated control cells inside the treatment zone where bats were excluded.

We found no evidence that excluding bats affected temporal changes in *P. destructans* prevalence in any site. We found no differences between swabs collected outside the bat exclosures and swabs from untreated control cells inside exclosures during the late winter period (Fig 4). Sample size was too low in the Alabama and Arkansas sites to assess if excluding bats affected fungal loads but, in the Ontario site, there was also no effect of excluding bats on fungal load (t(47.6) = 1.54, P > 0.1).

### Microbial diversity

**Fig 5:**
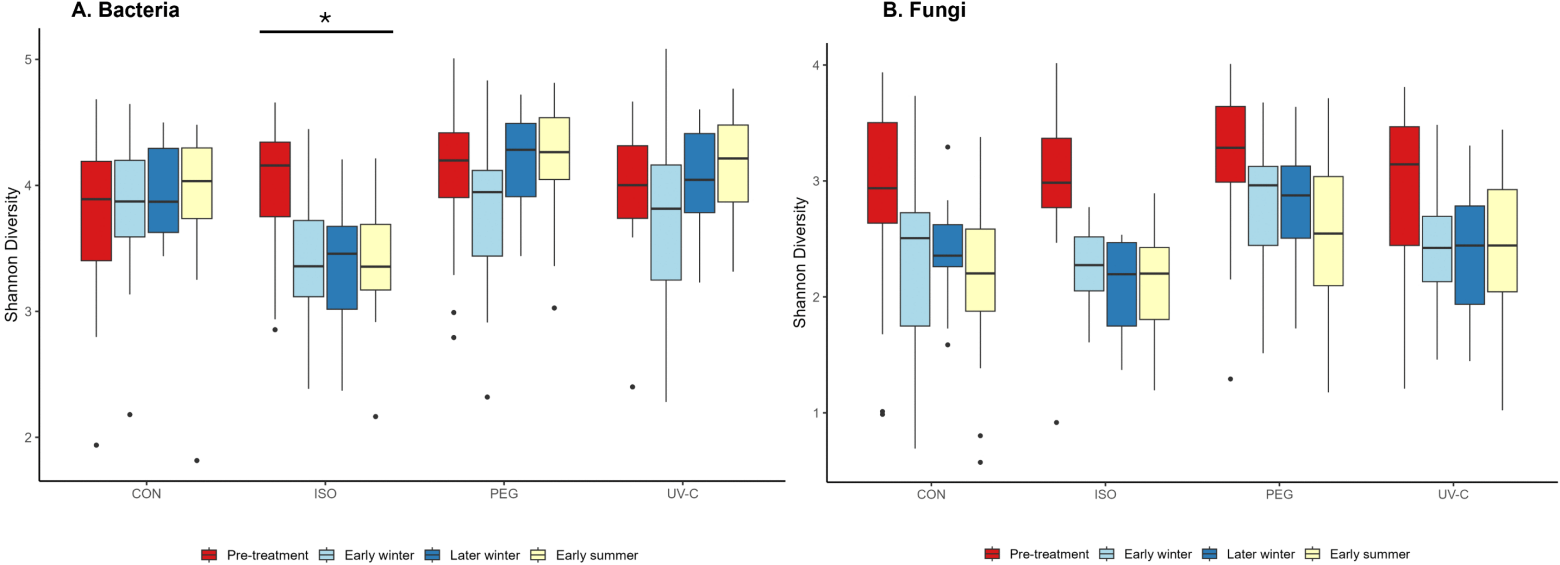
Shannon Diversity Index for bacterial taxa (A) and fungal taxa (B) in treatment cells in the hibernaculum in Ontario. The genus *Pseudosgymnoascus* was removed from fungal taxa diversity calculations because potential reductions in *P. destructans*, the targeted fungal species, would alter calculations of non-target effects. CON = control, ISO = isopropyl alcohol, PEG = polyethylene glycol, UV-C = ultraviolet-C radiation. Pre-treatment = “baseline” Shannon diversity before treatments occurred. The significant effect of treatment and time on bacterial diversity, driven solely by isopropyl alcohol (see results), is shown by the asterisk in **A.** The trend for declining fungal diversity with time was not significant.

Treatment and time significantly influenced Shannon diversity of bacteria (treatment: χ^2^ = 19.9, df = 3, *P* = 0.0002; time: χ^2^ = 8.8, df = 1, *P* = 0.003; S8 Table). However, this was driven by a decline in Shannon diversity over time in isopropyl treated cells (Fig 5A). Shannon diversity of fungi declined over time in all groups, but this change was not significant (χ^2^ = 3.54, df = 1, *P* = 0.06; Fig 5B). There was an effect of treatment on Shannon diversity (χ^2^ = 17.98, df = 3, *P* = 0.0004; S8 Table). However, this effect was due to a higher baseline diversity in the PEG group, not due to the treatment itself.

Principal Coordinate Analysis (PCoA) plots revealed weak clustering by the isopropyl treatment for both unweighted and weighted UniFrac distances for bacterial taxa, while PEG and UV-C exhibited less variation over time. Axes one and two explained 21.2% of the variation in unweighted UniFrac values and 38.9% of the variation in weighted UniFrac values. We found a significant effect of treatment on both unweighted (z = 3.51, *P* < 0.0001) and weighted UniFrac distances (z = 7.06, *P* < 0.0001). Treatment effects were driven by isopropyl application, but there was no significant effect of any other treatment (S9A Table). There was also an effect of time (z = −2.30, *P* = 0.021) on unweighted Unifrac values. Analysis of fungal sequences with PCoA showed weak clustering by isopropyl treatment for weighted but not unweighted UniFrac distances. Similar to bacteria, fungal OTUs showed minimal variation over time in PEG and UV-C treatments. Axes one and two explained 16.4% of the variation in unweighted and 36.2% of the variation in weighted UniFrac values. We found a significant effect of treatment, which was driven by isopropyl, on unweighted (z = 2.68, *P* = 0.007) UniFrac values (S9B Table) when comparing with baseline (pre-treatment) levels. There was, however, no effect of treatment on weighted UniFrac values. There were also no effects of any other treatment or time on fungal community composition.

## Discussion

Our results were not consistent with the hypothesis that a single application of UV-C light or PEG effectively reduces the environmental reservoir for *P. destructans*, as there was no decline of *P. destructans* on treated substrates in any of our sites. In the Ontario site, we did detect a decline in *P. destructans* load by early summer in cells treated with isopropyl. This result, however, was not consistent across sites and, unsurprisingly, isopropyl also negatively affected bacterial diversity in treatment cells suggesting that it would cause pronounced non-target effects.

The effectiveness of PEG and UV-C against *P. destructans* has been shown *in vitro* [26,28] and environmental application of PEG appeared to be effective at reducing both environmental transmission and WNS severity in captive and field experiments with live bats [17]. UV-C has also been shown to be effective in a whole-room *P. destructans* decontamination experiment in the laboratory [19]. Therefore, we were surprised to find no effect of either treatment in our field experiment. One explanation for the difference in our UV-C results compared to those of Kwait et al. [19] is that pathogen characteristics and/or environmental factors in real-world hibernacula limit the efficacy of UV-C relative to controlled laboratory conditions. We used double the effective PEG concentration and 4–10 times the effective UV-C dose identified as effective in the laboratory [17,19,26] but *P. destructans* growing in a field setting could be more resistant to treatments than lab-cultured fungus. Condensation on hibernaculum surfaces may have washed away PEG or isopropyl treatments, or the rough, uneven substrates in hibernacula may have caused uneven delivery of treatments, both of which could have resulted in remnant, post-treatment patches of viable *P. destructans* that then recolonized treated cells. We used many relatively small treatment cells to provide replication for our experiment and the small size of each cell could have further enabled *P. destructans* to recolonize from cell edges. However, the slow growth rate of *P. destructans* [53] means that other microbes would likely colonize treated surfaces more quickly.

Another explanation for our results is that we missed detecting treatment effects because non-viable *P. destructans* DNA had not yet degraded and was still present in our treatment cells by the time of sampling [16]. Kwait et al. [19] applied UV-C treatment directly to fungal cultures in their whole-room decontamination experiment and, ideally, we would have used fungal culture from swabs to assess viability of *P. destructans* following treatment. However, at present, there is no reliable culture viability assay for *P. destructans* at the low loads typical of natural hibernaculum substrates so we relied on the standard qPCR approach widely used for *P. destructans* surveillance and research [see 16 for discussion]. Unfortunately, there are also no data quantifying how long it takes *P. destructans* DNA to degrade in the environment in the absence of viable fungus, although it is assumed that DNA would be unlikely to persist for the weeks- to months-long intervals similar to those between our treatments and our sampling [16]. In our view, two important priorities for future research and management should be the development of culture techniques that are sensitive to the very small quantities of *P. destructans* typical of environmental substrates, and field experiments to determine the persistence of *P. destructans* DNA on hibernaculum substrates under different environmental conditions.

A final methodological explanation for the difference between our results and those of other trials could be sample size. Although we began the experiment with a large number of cells, site-wide *P. destructans* prevalence was only between 29–35% in the Alabama and Arkansas sites and decreased as the experiment progressed reducing our sample size further for the load analysis (S1 Table). Thus, a small sample size could have reduced our ability to detect treatment effects. Larger sample sizes, larger treatment cells, selection of sites with high initial fungal load, and a better method to assess *P. destructans* viability (including a culture method to test for *P. destructans* viability at low loads) [16] would all be useful for future studies.

Our results suggest that PEG and UV-C had minimal non-target effects on other microbiota at the Ontario site. This is more encouraging for the use of UV-C and PEG as treatment agents than our findings above, especially combined with other studies showing their effectiveness at reducing *P. destructans* in the environment and reducing WNS severity [17,19]. We did find that Shannon diversity of bacteria decreased over the experiment in isopropyl-treated cells, which was not surprising as isopropyl is a known antibacterial agent. Fungal diversity, on the other hand, slightly declined over time regardless of treatment group. This could reflect temperatures within the Ontario site, which could limit what fungal species can survive and when they can grow. Although overall fungal diversity decreased over the course of the experiment, *P. destructans* prevalence did not significantly change, suggesting that *P. destructans* is more tolerant of low temperatures than other resident fungi.

We observed a decline in *P. destructans* prevalence at all three sites, including in control cells, over the course of the experiment. Environmental factors, such as temperature and humidity, play a role in fungal and bacterial proliferation [31,32] and may have influenced the temporal changes we observed. Humidity fell below levels that caused relatively slow *P. destructans* growth in a laboratory culture study of the fungus (i.e., below 8.8 g/m^3^) [54] on almost half of the days between treatment and early winter sampling in the Alabama site (29/50). We were not able to record continuous humidity data for the other sites but absolute humidity in the Ontario site, between early winter and early summer sampling (Feb 20 – May 13), was also below a level, which would likely slow growth of *P. destructans* mycelia [54]. Temperatures during this time period also ranged between 0.85–5.95 °C, below the ideal growth temperatures for *P. destructans* [30]. These abiotic factors may have influenced *P. destructans* in the Ontario site and could explain the decline in *P. destructans* in our control cells. However, this begs the question how *P. destructans* was so widespread in this site before our experiment when environmental conditions did not appear to favour its growth. One possibility is that by excluding bats, presumably the ideal growth substrate for *P. destructans*, we made the habitat unsuitable enough for *P. destructans* that its growth and abundance declined, although we think this is unlikely (see below).

While cold temperatures may have influenced *P. destructans* growth in the Ontario site, warm temperatures may have impacted growth in Alabama and Arkansas. In the Alabama site, *P. destructans* prevalence surprisingly dropped to 0% across all cells, including controls, by early summer. This site was ~ 15 °C on average during the summer of our experiment and could reach higher temperatures in warmer years. Although 15 °C is within the optimal temperature range for *P. destructans* growth in laboratory studies [30], temperature effects have not been tested experimentally in field conditions and it is possible that high temperatures in the Alabama site allowed other microbes, more suited to higher temperatures, to outcompete *P. destructans*. Artificially adjusting hibernaculum microclimates in winter has been suggested as a way to reduce the potential for *P. destructans* to grow [55,56], but this could negatively affect the energy balance and survival of hibernating bats [57,58]. If high temperatures and reduced competitive ability of *P. destructans* explain elimination of *P. destructans* from our Alabama site during summer, this suggests potential for temporary microclimate manipulation, outside the hibernation season, when there would be no disturbance to bats, to help reduce environmental loads of *P. destructans*. Thus, in terms of research, we recommend additional testing of temporary microclimate manipulation during summer as an option to combat the environmental reservoir for *P. destructans.* We also recommend further testing of the treatments we tested in hibernacula with temperature and humidity conditions that consistently fall within the ideal range for *P. destructans* growth throughout the year. If the efficacy of these treatments can be confirmed in optimal hibernacula for *P. destructans*, it could make sense for conservation and management, to apply them in these kinds of sites but avoid investing resources in their use in hibernacula with sub-optimal conditions for *P. destructans*, or low *P. destructans* prevalence, where our experiment suggests they may contribute relatively little to additional control of the environmental reservoir.

Across sites, there was a consistent drop in *P. destructans* prevalence between pre-treatment and early winter in all groups. Most surprisingly, within control cells, prevalence dropped by as much as 82%. While temperature and humidity changes coinciding with the onset of winter likely influenced *P. destructans* prevalence, these results also led us to hypothesize that bat exclusion, and not treatments, were driving declines in *P. destructans* prevalence. We found no difference in prevalence inside versus outside the exclosures we built to exclude bats from the parts of hibernacula where we established treatment cells (Fig 4) which suggests that the absence of bats from inside the treatment zones does not explain the decline in prevalence we observed. Nevertheless, another priority for future research should be to repeat an experiment like ours without excluding bats from the treatment zone, to understand how the additional source of *P. destructans* from the bats themselves might influence treatment efficacy.

Another possible explanation for declines in *P. destructans* prevalence throughout winter is the “patchiness” of *P. destructans* on hibernaculum surfaces. Fungi are not uniformly distributed throughout the environment, as growth is influenced by water availability, mineral composition, and many other abiotic factors [31]. Before treatment application, *P. destructans* prevalence in the Arkansas and Alabama sites was 29–35%, indicating a patchy distribution throughout these hibernacula. While we chose experimental cells that were initially *P. destructans*-positive, *P. destructans* may not have been evenly distributed within each cell. It is therefore possible that variation of *P. destructans* prevalence within our experimental cells (i.e., *P. destructans* “patchiness”) contributed to the declines recorded in winter. Our swabbing method covered all quadrants every sampling period, so it is unlikely that uneven distribution of *P. destructans* within cells was the sole cause of decreasing prevalence over the winter. Nevertheless, even larger sample sizes may be needed to reduce impacts of patchy fungal distributions on results of treatment experiments.

Changes in *P. destructans* prevalence and load over the course of our study varied widely between the three study sites, which has implications for the design and implementation of environmental treatments. For example, prevalence in the Alabama site dropped to 0% by early summer but remained at ~75% in the Ontario site, which was not significantly different from levels in this site the preceding fall, despite the decline in *P. destructans* load that we observed in control cells for the Ontario site. This variation between sites, combined with the absence of treatment effects in our study compared to other studies of UV-C and PEG [17,19] suggests that patterns of persistence of *P. destructans*, and potential benefits of treatments, may vary among hibernacula. Future studies of environmental treatments, and management plans employing this approach, should consider this site-specific variability.

Encouragingly we found little evidence of non-target effects of PEG or UV-C when used as environmental treatments in bat hibernacula. Less encouraging, we found no evidence of significant effects of either treatment on *P. destructans* although aspects of our experimental design could have influenced our ability to detect an effect. As WNS continues to impact hibernating bat populations across North America, the success of environmental treatments may depend on identifying target hibernacula that most strongly contribute to the survival of at-risk species. Environmental treatments could then be developed for these target hibernacula while considering site-specific characteristics such as temperature, humidity, standing water presence, microbial composition and distribution, and other measurable abiotic factors.

## Supporting information

S1 Fig Swabbing scheme and timing for experimental cells. (PNG)

S1 Table Number of samples included in fungal load analysis at each site and sampling period. Cells were excluded from the load analysis during sampling periods in which *P. destructans* load was zero; therefore, the number of samples varies among treatments and across time. Pre-treatment samples reflect only those cells in which *P. destructans* was detected during the initial survey.
(PDF)

S2 Table Estimated model coefficients, standard errors, and significance of predictors for *P. destructans* prevalence in the Alabama site. This analysis includes a total of 35 cells that were *P. destructans*-positive during the pre-treatment period that were treated (PEG = 11; UV-C = 5; Isopropyl = 9; Untreated = 10) and then sampled three additional times. Due to singularity and lack of model convergence, a reduced full model was fit using the glm function (i.e., cell ID was not included as a random effect). The reference Treatment level was untreated control. The term Time reflects the number of days since pre-treatment sampling divided by 10. The coefficient and standard error associated with the term Location (Wall) reflects the effect of sampling cells on the wall as compared to sampling cells on the ceiling. The nested models used to conduct the likelihood ratio tests were fit using maximum likelihood.
(PDF)

S3 Table Estimated model coefficients, standard errors, and significance of predictors for *P. destructans* prevalence in the Arkansas site. This analysis includes a total of 42 cells that were *P. destructans*-positive during the pre-treatment period that were treated (PEG = 11; UV-C = 8; Isopropyl = 12; Untreated = 11) and then sampled three additional times. The model was fit with a binomial distribution with the glmer function from package lme4. Cell ID was included as a random effect. A model including the treatment:time interaction would not converge; therefore, this term was excluded from the full model. The significance of cell ID was tested using a likelihood ratio test comparing the full model to a binomial generalized linear model fit using the function glm (both models were fit using maximum likelihood). The proportion of variation explained by cell ID (*r*) was calculated by dividing the variance associated with cell ID by the total variance (cell ID variance + residual variance). The residual variance was assumed to be (π^2^)/3 (Nakagawa and Schielzeth 2010).
(PDF)

S4 Table Estimated model coefficients, standard errors, and significance of predictors for *P. destructans* prevalence in the Ontario site. This analysis includes a total of 110 cells that were *P. destructans-*positive during the pre-treatment period that were treated (PEG = 28; UV-C = 29; Isopropyl = 25; Untreated = 28) and then sampled three additional times. The model was fit with a binomial distribution with glmer function from package lme4. Cell ID was included as a random effect. The non-significant Treatment:Time interaction was removed from the full model. The coefficients, standard errors, significance, and proportion of variance explained by cell ID reflect values from a model excluding the Treatment:Time interaction. The nested models used to conduct the likelihood ratio tests were fit using maximum likelihood. The proportion of variation explained by cell ID (*r*) was calculated by dividing the variance associated with cell ID by the total variance (cell ID variance + residual variance). The residual variance was assumed to be (π^2^)/3 (Nakagawa and Schielzeth 2010).
(PDF)

S5 Table Estimated model coefficients, standard errors, and significance of predictors for log_10_ transformed *P. destructans* load in the Alabama site. The dataset for this analysis includes a total of nine *P. destructans* load values (PEG = 3; UV-C = 0; Isopropyl = 1; Untreated = 5) that were obtained subsequent to the pre-treatment period. The UV-C and Isopropyl treatments were removed from the dataset due to low sample sizes. The analysis was conducted using the function lm. We did not attempt to include a random effect of cell ID into the model because there was only one cell with multiple readings.
(PDF)

S6 Table Estimated model coefficients, standard errors, and significance of predictors for log_10_ transformed *P. destructans* load in the Arkansas site. The dataset for this analysis includes a total of 24 *P. destructans* load values (PEG = 4; UV-C = 6; Isopropyl = 9; Untreated = 5) that were obtained subsequent to the pre-treatment period. The model was fit with a Gaussian distribution using the lmer function from package lme4. Cell ID was included as a random effect. The non-significant Treatment:Time interaction was removed from the full model. The coefficients, standard errors, significance, and proportion of variance explained by cell ID reflect values from a model excluding the Treatment:Time interaction. Coefficients and standard errors reflect values from the model fit with restricted maximum likelihood. The nested models used to conduct the likelihood ratio tests were fit using maximum likelihood.
(PDF)

S7 Table Estimated model coefficients, standard errors, and significance of predictors for log_10_ transformed *P. destructans* load in the Ontario site. The dataset for this analysis includes a total of 256 *P. destructans* load values (PEG = 65; UV-C = 68; Isopropyl = 62; Untreated = 61) that were obtained subsequent to the pre-treatment period. The model was fit with a Gaussian distribution using the lmer function from package lme4. Cell ID was included as a random effect. Coefficients and standard errors reflect values from the model including the treatment:time interaction fit with restricted maximum likelihood. The nested models used to conduct the likelihood ratio tests were fit using maximum likelihood. NA values are provided for the significance of treatment and time because the presence of a significant treatment:time interaction makes these main effects misleading. The proportion of variation explained by cell ID (*r*) was calculated by dividing the variance associated with cell ID by the total variance (cell ID variance + residual variance).
(PDF)

S8 Table Results of linear mixed-effects models of the effects of treatment and time on the Shannon Diversity Index of bacterial operational taxonomic units (OTUs) (A) and non-target fungal OTUs (B). For bacterial diversity, Shannon Diversity Index values were log_10_ transformed prior to analysis. The model was fit using the lmer function from the package lme4, with cell ID included as a random effect. The nested models used to conduct the likelihood ratio tests were fit using maximum likelihood. The proportion of variation explained by cell ID (*r*) was calculated by dividing the variance associated with cell ID by the total variance (cell ID variance + residual variance).
(PDF)

S9 Table Results of linear mixed-effects models of the effects of treatment and time on the Weighted and Unweighted Unifrac Distances of bacterial operational taxonomic units (OTUs) (A) and non-target fungal OTUs (B). We used *qiime longitudinal first-distances* to calculate distances between the microbiome composition at the first recorded time point (pre-treatment) and subsequent time points for the same cell ID, based on unweighted and weighted UniFrac values.
(PDF)

S1 Data Stulberg et al 2026 - Data. (CSV)
