# Supplementary figures and images for: Ultraviolet light and polyethylene glycol as environmental cleaning agents to reduce contamination of Pseudogymnoascus destructans in bat hibernacula
Source: PLoS One. 2026 Jan 27;21(1):e0341213. doi: 10.1371/journal.pone.0341213 (PMC12843589; doi:10.1371/journal.pone.0341213)

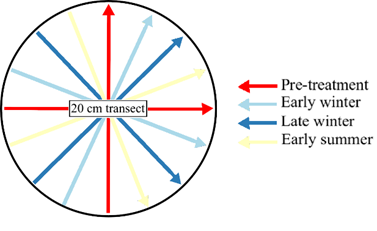

Supplement: S1 Fig — (PNG) [file pone.0341213.s001.png]
