# Supplementary material for: Ultraviolet light and polyethylene glycol as environmental cleaning agents to reduce contamination of Pseudogymnoascus destructans in bat hibernacula
Source: PLoS One. 2026 Jan 27;21(1):e0341213. doi: 10.1371/journal.pone.0341213 (PMC12843589; doi:10.1371/journal.pone.0341213)
Supplement: S1 Table — Cells were excluded from the load analysis during sampling periods in which P. destructans load was zero; therefore, the number of samples varies among treatments and across time. Pre-treatment samples reflect only those cells in which P. destructans was detected during the initial survey. (PDF) [file pone.0341213.s002.pdf]

A. Ontario	Pre-treatment	Early winter	Late winter	Early summer
CON	28	18	25	25
ISO	25	19	22	24
PEG	28	15	25	24
UV-C	29	20	26	24

B. Alabama	Pre-treatment	Early winter	Late winter	Early summer
CON	10	2	4	1
ISO	9	0	1	0
PEG	11	2	1	0
UV-C	5	0	2	0

C. Arkansas	Pre-treatment	Early winter	Late winter	Early summer
CON	11	2	2	1
ISO	12	4	3	2
PEG	11	3	0	1
UV-C	8	2	4	1
