# Supplementary material for: Ultraviolet light and polyethylene glycol as environmental cleaning agents to reduce contamination of Pseudogymnoascus destructans in bat hibernacula
Source: PLoS One. 2026 Jan 27;21(1):e0341213. doi: 10.1371/journal.pone.0341213 (PMC12843589; doi:10.1371/journal.pone.0341213)
Supplement: S2 Table — This analysis includes a total of 35 cells that were P. destructans-positive during the pre-treatment period that were treated (PEG = 11; UV-C = 5; Isopropyl = 9; Untreated = 10) and then sampled three additional times. Due to singularity and lack of model convergence, a reduced full model was fit using the glm function (i.e., cell ID was not included as a random effect). The reference Treatment level was untreated control. The term Time reflects the number of days since pre-treatment sampling divided by 10. The coefficient and standard error associated with the term Location (Wall) reflects the effect of sampling cells on the wall as compared to sampling cells on the ceiling. The nested models used to conduct the likelihood ratio tests were fit using maximum likelihood. (PDF) [file pone.0341213.s003.pdf]

	Coefficient	Std. error	χ^2	DF	P-value
**Treatment**			5.9	3	0.12
PEG	-0.73	0.80			
UV-C	-17.0	1630			
Isopropyl	-1.71	1.15			
**Time**	-0.09	0.05	3.3	1	0.07
**Location (Wall)**	0.10	0.74	0.02	1	0.89
