# Supplementary material for: Ultraviolet light and polyethylene glycol as environmental cleaning agents to reduce contamination of Pseudogymnoascus destructans in bat hibernacula
Source: PLoS One. 2026 Jan 27;21(1):e0341213. doi: 10.1371/journal.pone.0341213 (PMC12843589; doi:10.1371/journal.pone.0341213)
Supplement: S3 Table — This analysis includes a total of 42 cells that were P. destructans-positive during the pre-treatment period that were treated (PEG = 11; UV-C = 8; Isopropyl = 12; Untreated = 11) and then sampled three additional times. The model was fit with a binomial distribution with the glmer function from package lme4. Cell ID was included as a random effect. A model including the treatment:time interaction would not converge; therefore, this term was excluded from the full model. The significance of cell ID was tested using a likelihood ratio test comparing the full model to a binomial generalized linear model fit using the function glm (both models were fit using maximum likelihood). The proportion of variation explained by cell ID (r) was calculated by dividing the variance associated with cell ID by the total variance (cell ID variance + residual variance). The residual variance was assumed to be (π2)/3 (Nakagawa and Schielzeth 2010). (PDF) [file pone.0341213.s004.pdf]

	Coefficient	Std. error	χ^2	DF	P-value
**Treatment**			1.7	3	0.64
PEG	-0.25	1.02			
UV-C	0.78	1.03			
Isopropyl	0.77	0.94			
**Time**	-0.07	0.04	3.57	1	0.06
**Location (Wall)**	-0.15	0.74	0.04	1	0.84
**Cell (Random)**	$r = 0.36$		48.3	1	< 0.0001
