# Supplementary material for: Ultraviolet light and polyethylene glycol as environmental cleaning agents to reduce contamination of Pseudogymnoascus destructans in bat hibernacula
Source: PLoS One. 2026 Jan 27;21(1):e0341213. doi: 10.1371/journal.pone.0341213 (PMC12843589; doi:10.1371/journal.pone.0341213)
Supplement: S4 Table — This analysis includes a total of 110 cells that were P. destructans-positive during the pre-treatment period that were treated (PEG = 28; UV-C = 29; Isopropyl = 25; Untreated = 28) and then sampled three additional times. The model was fit with a binomial distribution with glmer function from package lme4. Cell ID was included as a random effect. The non-significant Treatment:Time interaction was removed from the full model. The coefficients, standard errors, significance, and proportion of variance explained by cell ID reflect values from a model excluding the Treatment:Time interaction. The nested models used to conduct the likelihood ratio tests were fit using maximum likelihood. The proportion of variation explained by cell ID (r) was calculated by dividing the variance associated with cell ID by the total variance (cell ID variance + residual variance). The residual variance was assumed to be (π2)/3 (Nakagawa and Schielzeth 2010). (PDF) [file pone.0341213.s005.pdf]

	Coefficient	Std. error	χ^2	DF	P-value
**Treatment**			1.9	3	0.59
PEG	0.29	0.47			
UV-C	0.37	0.47			
Isopropyl	0.68	0.50			
**Time**	0.06	0.02	9.8	1	0.002
**Location (Wall)**	0.05	0.35	0.02	1	0.89
**Treatment:Time**			1.3	3	0.73
**Cell (Random)**	$r = 0.21$		4.9	1	0.03
