# Supplementary material for: Ultraviolet light and polyethylene glycol as environmental cleaning agents to reduce contamination of Pseudogymnoascus destructans in bat hibernacula
Source: PLoS One. 2026 Jan 27;21(1):e0341213. doi: 10.1371/journal.pone.0341213 (PMC12843589; doi:10.1371/journal.pone.0341213)
Supplement: S5 Table — The dataset for this analysis includes a total of nine P. destructans load values (PEG = 3; UV-C = 0; Isopropyl = 1; Untreated = 5) that were obtained subsequent to the pre-treatment period. The UV-C and Isopropyl treatments were removed from the dataset due to low sample sizes. The analysis was conducted using the function lm. We did not attempt to include a random effect of cell ID into the model because there was only one cell with multiple readings. (PDF) [file pone.0341213.s006.pdf]

	Coefficient	Std. error	F	DF	p-value
**Treatment**					
PEG	0.51	0.41	1.5	1,6	0.26
