# Supplementary material for: Ultraviolet light and polyethylene glycol as environmental cleaning agents to reduce contamination of Pseudogymnoascus destructans in bat hibernacula
Source: PLoS One. 2026 Jan 27;21(1):e0341213. doi: 10.1371/journal.pone.0341213 (PMC12843589; doi:10.1371/journal.pone.0341213)
Supplement: S6 Table — The dataset for this analysis includes a total of 24 P. destructans load values (PEG = 4; UV-C = 6; Isopropyl = 9; Untreated = 5) that were obtained subsequent to the pre-treatment period. The model was fit with a Gaussian distribution using the lmer function from package lme4. Cell ID was included as a random effect. The non-significant Treatment:Time interaction was removed from the full model. The coefficients, standard errors, significance, and proportion of variance explained by cell ID reflect values from a model excluding the Treatment:Time interaction. Coefficients and standard errors reflect values from the model fit with restricted maximum likelihood. The nested models used to conduct the likelihood ratio tests were fit using maximum likelihood. (PDF) [file pone.0341213.s007.pdf]

	Coefficient	Std. error	χ^2	DF	P-value
**Treatment**			5.2	3	0.16
PEG	-0.25	0.49			
UV-C	0.66	0.45			
Isopropyl	0.07	0.41			
**Time**	-0.007	0.02	0.05	1	0.83
**Location (Wall)**	-0.10	0.32	0.13	1	0.72
**Treatment:Time**			0.56	3	0.90
**Cell (Random)**			0.24	1	0.62
