# Supplementary material for: Ultraviolet light and polyethylene glycol as environmental cleaning agents to reduce contamination of Pseudogymnoascus destructans in bat hibernacula
Source: PLoS One. 2026 Jan 27;21(1):e0341213. doi: 10.1371/journal.pone.0341213 (PMC12843589; doi:10.1371/journal.pone.0341213)
Supplement: S7 Table — The dataset for this analysis includes a total of 256 P. destructans load values (PEG = 65; UV-C = 68; Isopropyl = 62; Untreated = 61) that were obtained subsequent to the pre-treatment period. The model was fit with a Gaussian distribution using the lmer function from package lme4. Cell ID was included as a random effect. Coefficients and standard errors reflect values from the model including the treatment:time interaction fit with restricted maximum likelihood. The nested models used to conduct the likelihood ratio tests were fit using maximum likelihood. NA values are provided for the significance of treatment and time because the presence of a significant treatment:time interaction makes these main effects misleading. The proportion of variation explained by cell ID (r) was calculated by dividing the variance associated with cell ID by the total variance (cell ID variance + residual variance). (PDF) [file pone.0341213.s008.pdf]

	Coefficient	Std. error	χ^2	DF	P-value
**Treatment**					NA
PEG	-0.20	0.21			
UV-C	0.12	0.21			
Isopropyl	0.26	0.21			
**Time**	0.004	0.008			NA
**Location (Wall)**	-0.28	0.08	11.6	1	0.0007
**Treatment:Time**			9.0	3	0.03
**Cell (Random)**	$r = 0.30$		16.1	1	< 0.0001
