# Supplementary material for: Ultraviolet light and polyethylene glycol as environmental cleaning agents to reduce contamination of Pseudogymnoascus destructans in bat hibernacula
Source: PLoS One. 2026 Jan 27;21(1):e0341213. doi: 10.1371/journal.pone.0341213 (PMC12843589; doi:10.1371/journal.pone.0341213)
Supplement: S8 Table — For bacterial diversity, Shannon Diversity Index values were log10 transformed prior to analysis. The model was fit using the lmer function from the package lme4, with cell ID included as a random effect. The nested models used to conduct the likelihood ratio tests were fit using maximum likelihood. The proportion of variation explained by cell ID (r) was calculated by dividing the variance associated with cell ID by the total variance (cell ID variance + residual variance). (PDF) [file pone.0341213.s009.pdf]

**A. Bacteria**	Coefficient	Std. error	χ^2	DF	P-value
**Treatment**			19.9	3	0.0002
PEG	-0.04	0.03			
UV-C	-0.005	0.03			
Isopropyl	0.09	0.03			
**Time**			8.8	1	0.003
**Location (Wall)**			0.54	1	0.46
**Treatment:Time**			5.7	3	0.13
**Cell (Random)**	*r* = 0.58		54.3	7	< 0.001

**B. Fungi**	Coefficient	Std. error	χ^2	DF	P-value
**Treatment**			17.98	3	0.0004
PEG	0.43	0.13			
UV-C	0.21	0.13			
Isopropyl	-0.10	0.13			
**Time**			3.54	1	0.06
**Location (Wall)**			3.51	1	0.06
**Treatment:Time**			1.38	3	0.71
**Cell (Random)**	*r* = 0.43		27.68	7	0.0003
