# Supplementary material for: Ultraviolet light and polyethylene glycol as environmental cleaning agents to reduce contamination of Pseudogymnoascus destructans in bat hibernacula
Source: PLoS One. 2026 Jan 27;21(1):e0341213. doi: 10.1371/journal.pone.0341213 (PMC12843589; doi:10.1371/journal.pone.0341213)
Supplement: S9 Table — We used qiime longitudinal first-distances to calculate distances between the microbiome composition at the first recorded time point (pre-treatment) and subsequent time points for the same cell ID, based on unweighted and weighted UniFrac values. (PDF) [file pone.0341213.s010.pdf]

**A. Bacteria**	Coefficient	Std. error	Z-value	P-value
**Unweighted Unifrac Distances**				
Intercept	0.536	0.018	29.76	<0.0001
PEG	-0.049	0.025	-1.94	0.052
UV-C	-0.019	0.031	-0.618	0.537
Isopropyl	0.092	0.026	3.51	<0.0001
Time	-0.000	0.000	-2.30	0.021
Time:PEG	0.000	0.000	1.27	0.204
Time:UV-C	0.000	0.000	1.37	0.171
Time:Isopropyl	0.000	0.000	0.802	0.423
Group Variance	0.002	0.015		
**Weighted Unifrac Distances**				
Intercept	0.164	0.013	12.60	<0.001
PEG	0.004	0.018	0.211	0.833
UV-C	0.019	0.022	0.856	0.392
Isopropyl	0.136	0.019	7.06	<0.0001
Time	-0.000	0.000	-0.066	0.948
Time:PEG	0.000	0.000	0.271	0.786
Time:UV-C	0.000	0.000	0.449	0.654
Time:Isopropyl	-0.000	0.000	-0.113	0.910
Group Variance	0.002	0.020		

B. Fungi	Coefficient	Std. error	F-value	DF	P-value
**Unweighted Unifrac Distances**					
Intercept	0.551	0.024	23.39	<0.0001	
PEG	0.006	0.032	0.194	0.846	
UV-C	0.017	0.032	0.535	0.593	
Isopropyl	0.076	0.032	2.38	0.018	
Time	0.000	0.000	0.95	0.342	
Time:PEG	0.000	0.000	0.405	0.685	
Time:UV-C	-0.000	0.000	-0.648	0.517	
Time:Isopropyl	0.000	0.000	0.245	0.800	
Group Variance	0.003	0.019			
**Weighted Unifrac Distances**					
Intercept	0.461	0.058	7.89	<0.0001	
PEG	-0.019	0.079	-0.236	0.813	
UV-C	0.024	0.079	0.297	0.766	
Isopropyl	0.155	0.079	1.95	0.051	
Time	-0.000	0.000	-0.845	0.398	
Time:PEG	0.000	0.000	0.923	0.356	
Time:UV-C	0.000	0.000	0.025	0.980	
Time:Isopropyl	0.000	0.000	0.162	0.871	
Group Variance	0.027	0.058			
